# TRMT6 promotes hepatocellular carcinoma progression through the PI3K/AKT signaling pathway

**DOI:** 10.1186/s40001-022-00951-1

**Published:** 2023-01-27

**Authors:** Yanqing Ye, Maosheng Liu, Fengfei Wu, Shiyu Ou, Weidong Wang, Jieying Fei, Fang Xie, Lan Bai

**Affiliations:** 1 Guangdong Provincial Key Laboratory of Gastroenterology, Department of Gastroenterology, Nanfang Hospital, Southern Medical University, 1838 North Guangzhou Avenue, Guangzhou, 510515 Guangdong People’s Republic of China; 2 Department of Gastroenterology, The First Affiliated Hospital of Gannan Medical University, Ganzhou, 341000 Jiangxi People’s Republic of China; 3 Department of Gastroenterology, The Fourth Affiliated Hospital of Guangxi Medical University, Liuzhou, 545005 Guangxi People’s Republic of China

**Keywords:** Hepatocellular carcinoma, Cell proliferation, TRMT6, PI3K/AKT, mTOR

## Abstract

**Background:**

Hepatocellular carcinoma is one of the most common and deadly cancers. The aim of this study was to elucidate the role of tRNA methyltransferase 6 (TRMT6) during HCC progression.

**Methods:**

The role of TRMT6 in the progression and prognosis of HCC was confirmed by analysis of online databases and clinical human samples. The effects of up-regulation or down-regulation of TRMT6 on HCC cell proliferation and PI3K/AKT pathway-related protein expressions were verified. The molecular mechanism was investigated in vivo by constructing subcutaneous xenograft tumor model.

**Results:**

TRMT6 was overexpressed in HCC tissues and associated with Tumour-Node-Metastasis (TNM) stage, primary tumor (T) and regional lymph node (N) classification. TRMT6 expressions in HCC cell lines were higher than that in normal liver cell. TRMT6 overexpression can promote HCC cell proliferation, increase the number of S phase cells. Interference with TRMT6 reduced the PI3K/AKT pathway-related protein expressions, and was reversed by the addition of IGF1. Interference with TRMT6 inhibited tumor growth in vivo and was related to PI3K/AKT pathway.

**Conclusions:**

Overexpression of TRMT6 promote HCC cell proliferation in vivo and in vitro through PI3K/AKT/mTOR axis, which provides a potential choice for the treatment of HCC in clinical practice.

**Supplementary Information:**

The online version contains supplementary material available at 10.1186/s40001-022-00951-1.

## Introduction

Hepatocellular carcinoma (HCC) is one of the most common primary liver tumors in the world. It is characterized by high morbidity, rapid growth, strong invasion and high mortality [1–3]. Surgery is the most common method of treatment of liver cancer, but it is not appropriate for most HCC patients present with advanced stage. Even though, progress has been made in the surgical and medical treatment of liver cancer, the prognosis of most liver cancer patients worldwide is still poor [4]. Therefore, it is urgent to find more effective diagnostic and prognostic biomarkers for HCC to address this issue. Our study aims to further elucidate the molecular mechanisms underlying HCC carcinogenesis and progression for the development of new diagnostic methods and the identification of new therapeutic targets [5].

In recent years, with the rapid development of high-throughput sequencing technology, epigenetic modification has received more attention [6]. The epigenetic modification include DNA methylation, histone modifications and RNA methylation, etc. [6, 7]. There are more than 100 kinds of post-transcriptional modifications of RNA molecules [8, 9], among which methylation is the most common and mainly regulates gene expression at the post-transcriptional level [10]. RNA methylation can occur in all types of RNA including messenger RNA (mRNA), transfer RNA (tRNA), ribosomal RNA (rRNA) and non-coding RNA [11]. The most common RNA methylation modification is the methylation of N6 adenosine (m6A), which has been shown to be associated with the occurrence and development of many tumors [12].

Translation initiation is a rate-limiting step of gene expression, and tRNA_i_^Met^ plays a very important role in the recognition of initiation codon and the initiation of translation [13–15]. The expression level of tRNA_i_^Met^ is increased in tumors and may promotes the progression of the disease [13]. tRNA methyltransferase 6 (TRMT6)/TRMT61A is a methylase complex composed of the catalytic subunit TRMT61A and the binding subunit TRMT6, which is responsible for the m^1^A58 modification of tRNA and can specifically identify the m^1^A site in the 5’UTR sequence of mRNA that conforms to the “GUUCRA” motif [16, 17]. Studies have confirmed that TRMT6/61A is abnormal in bladder cancer cells, and silencing the expression of TRMT61A in bladder cancer cells can inhibit cell proliferation and invasion and promote cell apoptosis [18]. Our previous database analysis confirmed that TRMT6/TRMT61A, a methylase complex composed of the catalytic subunit TRMT61A and the binding subunit TRMT6, is responsible for m^1^A58 modification of tRNA and can specifically identify m^1^A sites in the 5’UTR sequence of mRNA that conforms to the “GUUCRA” motif [16, 17], and was closely related to the progression and prognosis of HCC patients. However, the specific mechanism by which TRMT6 expression regulates hepatocarcinogenesis and HCC progression has not been reported.

In conclusion, this study intends to clarify the specific functions of TRMT6 in the occurrence and development of HCC, which may be a new potential therapeutic target for HCC.

## Materials and methods

### Gene expression analysis and prognosis analysis of TRMT6

The Cancer Genome Atlas (TCGA) database (https://portal.gdc.cancer.gov/repository) was used to analyze the differential expression of *TRMT6* mRNA in liver hepatocellular carcinoma (HCC) tissues (*n* = 369) and paracancerous tissues (*n* = 50). The Human Protein Atlas (HPA) online database (https://www.proteinatlas.org/ENSG00000089195-TRMT6/pathology/liver+cancer), a comprehensive human proteomics portal, was used to perform TRMT6-related survival analysis.

### Clinical samples

All patients (*N* = 76) participating in the experiment signed informed consent, and the study was approved by the Clinical Ethics Committee of Southern Medical University. HCC tissue microarray containing 76 tumor tissues and corresponding paracancerous tissue specimens were purchased from Superbiotek (Shanghai, China). All the 76 cases of cancer tissues were confirmed to be HCC tissue by postoperative pathology, and all the adjacent tissue specimens were normal tissues without tumor cell invasion. All the 76 cases have complete clinical data. Among the 76 HCC patients, there were 69 males and 7 females, ˂ 50 years were 40 cases and ≥ 50 years were 36 cases. Liver cancer staging is based on the Tumour-Node-Metastasis (TNM) classification developed by the World Health Organization, including I (22 cases), II (18 cases), III (19 cases) and IV (17 cases) [19]. Primary tumor (T) classification contains T1 (22 cases), T2 (27 cases), T3 (20 cases) and T4 (7 cases), Regional lymph node (N) classification contains N0 (70 cases) and N1 (6 cases). Distant metastasis (M) classification contains M0 (59 cases) and M1 (17 cases).

### Immunohistochemical staining (IHC)

The paraffin-embedded tissue samples of patients in both groups were obtained. TRMT6 and Ki-67 protein was determined by IHC staining [20]. After washing paraffin sections for several times, milk liquid was added to block the antibody for 5 min. TRMT6 protein antibody (1:100, ab235321, abcam) or Ki-67 protein antibody (1:200, ab16667, abcam) was added and incubated at 4 ℃ for 2 h. After rinsing with phosphate buffer solution (PBS) for 3 times, the fluorescent stained secondary antibody was added, incubated at 37 ℃ for 30 min, and washed with PBS. Then, diaminobenzidine (DAB) was added, and the sections were re-stained with hematoxylin, dehydrated by gradient ethanol, sealed with neutral gum, and observed under confocal laser scanning microscope (Leica).

The IHC score of TRMT6 was defined according to Genecard (https://www.genecards.org/) and HPA database, which showed that the sublocation of TRMT6 was mainly in the nucleus and cytoplasm. Thus, the IHC score of TRMT6 was defined as nuclear IHC score + cytoplasmic IHC score. The following table is the criterion of integral judgment.

### Cell culture and treatment

Human hepatic stellate cells LX-2 was purchased from Millipore. LX-2 cells were cultured with 1640 high glucose containing 10% fetal bovine serum (FBS), 100 U/mL penicillin and 100 μg/mL streptomycin in a 5% CO_2_ incubator at 37 ℃, and the culture medium was changed every 24 h. Human hepatocellular carcinoma cell line HepG2, Huh-7, SMMC-7721, MHCC97L, MHCC97H were both purchased from ATCC (American type culture collection). These cells were cultured in DMEM medium containing 10% FBS, 100 U/mL penicillin and 100 μg/mL streptomycin at 37℃ and 5% CO_2_ in a constant temperature incubator. All the above cells were subcultured when the cells were fused to 80%–90%, and the logarithmic growth phase cells were taken for the experiment.

The short hairpin RNA (shRNA) targeting TRMT6 (sh*TRMT6*, 5′-GGGAAAGTTCTGAGTATTTAT-3′) and pcDNA 3.1-*TRMT6* (F: 5′-CGGCATCCATGGAGGGCTCAGGGGAG-3′; R: 5′-CCGCTCGAGTTAAGAGTCAGACTCTGGGCATTTTCGTTTTTTAGCTG-3′) were designed and synthesized by Shanghai Gemma Pharmaceutical Technology Co., Ltd. HepG2 cells were inoculated in 6-well plates and cultured in DMEM culture medium, and then grouped into control group, overexpressed (OE)-TRMT6 group and shTRMT6 group. The 250 μL medium was premixed with 10 μL (200 pmol) shRNA. In addition, another 250 μL medium was mixed with 5 μL Lipofectamine^™^ 2000 kit (Invitrogen), and incubated at room temperature for 5 min. Next, the above two mixtures were gently mixed and incubated at room temperature for 20 min. Then, they were added to 6-well plates and cultured in a damp incubator at 37 ℃ and 5% CO_2_. The cells were collected 48 h after transfection for further study. Three repeat wells were set up in each group. QRT-PCR and Western blot were used to detect the silencing/overexpression efficiency. After transfected with sh*TRMT6* 48 h later, HepG2 cells were treated with 100 ng/mL insulin-like growth factor 1 (IGF1; Promega) for 1 h, followed by subsequent experimental analysis. The experiments were grouped into control group, sh*TRMT6* group and sh*TRMT6* + IGF1 group.

### QRT-PCR

The total RNA samples in the LX2, HepG2, Huh-7, SMMC-7721, MHCC97L and MHCC97H cells in logarithmic growth phase were extracted with Trizol reagent (Invitrogen). RNA was reverse-transcribed into cDNA using HiFiScript cDNA Synthesis Kit (CWBIO, https://www.cwbio.com/news/aboutus.html), and the reaction conditions were 37 ℃ for 15 min, 85 ℃ for 5 s. The reaction solution was prepared according to the instructions of the FastSYBR Mixture reaction kit (CWBIO). The relative expression of TRMT6 is calculated by 2^−∆∆Ct^, and GAPDH was used as an internal reference.

### Western blot

LX2 and HCC cells in logarithmic growth stage were washed once with precooled PBS, and then lysed on the ice box with RIPA protein lysate (including PMSF protease inhibitor). The collected tumor tissues were frozen and crushed by liquid nitrogen and then cleaved by RIPA. After centrifugation at 12,000 r/min at 4 ℃ for 20 min, the protein supernatant was taken for quantification with the BCA protein concentration determination kit. After adding 5 × loading buffer, the protein was heated at 100℃ for 5 min for denaturation. Then, SDS–PAGE electrophoresis was performed. The protein was transferred to polyvinylidene fluoride (PVDF) membrane by wet transfer method, and sealed with 5% skim milk powder for 1.5 h. Then, they were washed with TBST buffer for 3 times, 10 min each time, and the primary antibodies from abcam (anti-TRMT6, 1:1000, ab235321; anti-p-PI3K, 0.5 µg/mL, ab182651; anti-p-AKT, 1:500, ab38449; anti-p-mTOR, 1:10,000, ab109268) were added, respectively, and incubated overnight at 4 °C. On the second day, after PVDF membrane was incubated for 15 min at room temperature, the membrane was washed with TBST buffer for 3 times, and incubated with corresponding secondary antibodies for 1 h at room temperature. After washing the membrane with TBST buffer for 3 times, the film was developed with ECL Chemiluminescence Kit (Beyotime) [35]. The gray value of the bands was analyzed with Image J 1.8.0 software. ACTB was used as internal reference to calculate the relative protein expression.

### Cell proliferation and viability assay

CCK-8 assay was performed to determine the number of living cells during cell proliferation. The HepG2 cells were inoculated in 96-well plates at a density of 5 × 10^3^/well. After transfection for 24, 48, 72 or 96 h, 10 μL CCK-8 reagent (Transgen Biotech Co., LTD) was added to each well and incubated at 37 ℃ for 1 h. Measure the absorbance at 450 nm according to the instructions.

EdU (5-Ethynyl-2′-deoxyuridine) assay was performed to detect HepG2 cells in the dividing phase (S phase) and analyze HepG2 cell proliferation using BeyoClick^TM^EdU-488 Cell Proliferation Kit (Beyotime). The density of HepG2 cells was adjusted, and 5 × 10^3^ per well were spread in a 24-well plate. After transfection for 24 h, EDU reagent was mixed into the culture medium for 12 h. After fluorescence staining, the cell density was observed under a fluorescence microscope.

### Cell cycle distribution assay

Flow cytometry was performed to analyze cell cycle of HepG2 cell transfected with pcDNA-*TRMT6* or sh*TRMT6*. HepG2 cells in logarithmic growth phase were seeded into 6-well plates with 1 × 10^5^ cells per well, and then cultured for 48 h. The cells were collected, fixed with anhydrous alcohol and stained overnight at 4 ℃ with propidium iodide PI (10 μg/mL; Sigma) for 30 min. The cells were washed with pre-cooled PBS buffer solution. Cell cycle analysis was performed by flow cytometry (EPICS XL-4; Beckman).

### Construction of subcutaneous xenograft tumor model

After transfected with sh*TRMT6* or pcDNA 3.1-*TRMT6*, HepG2 cells in logarithmic growth phase were digested with trypsin and counted 12 h before transfection. Cells were seeded into 6-well plates with a cell count of about 6 × 10^5^ per well. Transfection was carried out when the cell fusion degree reached about 20% under the condition of MOI = 10. The construction and packaging of TRMT6 overexpression/interference lentivirus was completed by Shanghai Genechem Co., Ltd. Male BALB/c nude mice aged 6–7 weeks were purchased from Vital River Co., Ltd. The animal experiments were supported by the Animal Care and Use Committee of Southern Medical University. HepG2 cells during logarithmic growth phase were prepared into cell suspension, and the cell count was adjusted to 2 × 10^6^ cells/mL, and then injected into the right subcutaneous back of nude mice (dose: 0.2 mL/animal; *n* = 6). The mice were grouped into control, OE-*TRMT6* and sh*TRMT6*. Four weeks later, the nude mice were euthanized, and tumor tissues were collected for tumor volume detection and Western blot detection of PI3K, AKT, mTOR.

### Statistical analysis

The t test was used to compare the measurement data of normal distribution between two groups. The measurement data of non-normal distribution were compared between the two groups using Wilcoxon signed rank test. Chi-square test was used to compare whether TRMT6 was statistically significant with clinicopathological features. The prognosis was analyzed by Kaplan–Meier survival analysis. The Spearman correlation coefficient was used to analyze the correlation. SPSS 20.0 statistical software was used to calculate, and *P* < 0.05 was considered statistically significant.

## Results

### Expression of TRMT6 in HCC tissues and cells

**Fig. 1:**
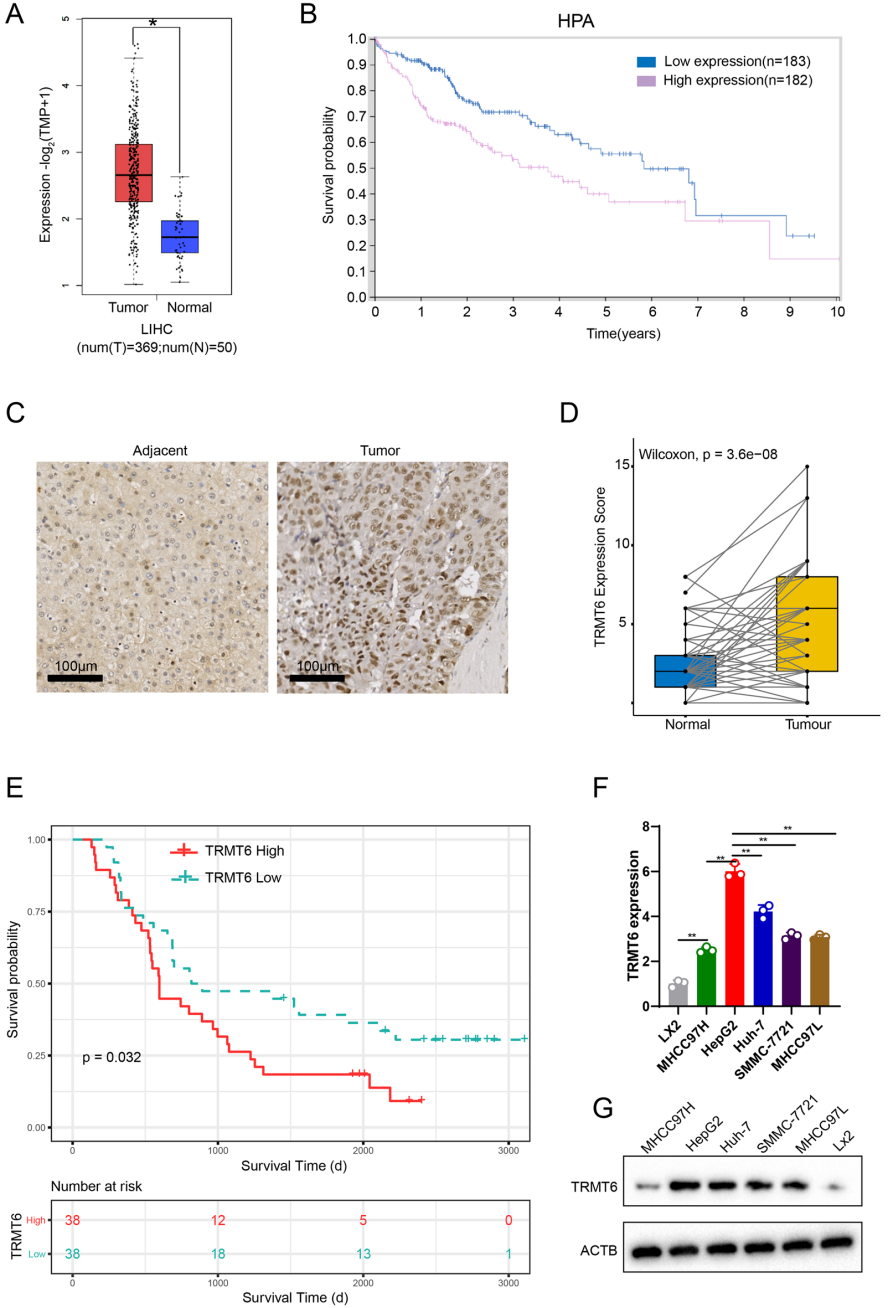
Expressions of TRMT6 in HCC tissues and cells. **A** Expressions of TRMT6 gene in LIHC cancer tissues (*n* = 369) and paracancerous tissues (*n* = 50) in the TCGA-LIHC data set were analyzed. **B** HPA database was used to perform TRMT6-related survival analysis. P (0.032) < 0.05. **C**, **D** Tissue microarray analysis was used to analyze IHC data of TRMT6 in HCC tissues (*n* = 76) and paracancerous tissues (*n* = 76). *P* < 0.05; Wilcoxon signedrank test. **E** Using Kaplan–Meier curve to analyze the correlation between TRMT6 and HCC prognosis. **F**, **G** mRNA and protein expressions of TRMT6 in the normal liver cell line and HCC cell lines were detected by qRT-PCR and Western blot assay. *P* < 0.01

According to the LIHC data set in the TCGA database, the expressions of TRMT6 gene in LIHC cancer tissues (*n* = 369) and paracancerous tissues (*n* = 50) were statistically analyzed, and it was found that TRMT6 was highly expressed in cancer tissues in the TCGA–LIHC data set (Fig. 1A). Survival analysis was performed using the HPA database, and the results showed that the survival time of HCC patients with high TRMT6 expression (*n* = 182) was significantly lower than that of HCC patients with low TRMT6 expression (*n* = 183) (Fig. 1B). Normal paracancerous tissues (*n* = 76) and HCC tissues (*n* = 76) were collected for microarray analysis. As shown in the Fig. 1C, IHC assay showed that the staining intensity of TRMT6 in Tumor group was significantly higher than that in Adjacent group. To further detect TRMT6 protein expression, the IHC score of TRMT6 in normal paracancerous tissues and HCC tissues was evaluated according to integral criterion (Table 1). Since the IHC score did not conform to the normal distribution, Wilcoxon signed rank test of paired samples was conducted, and statistical analysis showed that TRMT6 was significantly overexpressed in HCC tissues (Fig. 1D). Next, we divided the microarray samples into two groups with high TRMT6 expression and low TRMT6 expression according to the median of IHC score. Results of Kaplan–Meier survival analysis showed that high TRMT6 expression may be a significant risk factor affecting the prognosis of HCC (Fig. 1E). Furthermore, a statistical analysis of TRMT6 and related clinical information was performed (Table 2), and the results showed that TRMT6 was associated with TNM stage, T and N classification. Spearman’s rank correlation coefficient analysis was performed between the scores of Ki67 and TRMT6, and the results showed that they were positively correlated (Additional file 1: Figure S1). The mRNA and protein expressions of TRMT6 in the normal liver cell line LX-2 and HCC cell lines (HepG2, Huh-7, SMMC-7721, MHCC97L and MHCC97H) were detected by qRT-PCR and Western blot assays. The expression levels of TRMT6 in all HCC cell lines were higher than that in normal liver cell, and the highest expression was found in HepG2 cells (Fig. 1F, G).

**Table 1:** Criteria for judging immunohistochemical scores

Proportion of positive cells	< 5%	5–25%	26–50%	51–75%	76–100%
Point	0	1	2	3	4
Staining strength of positive cells	colourless	faint yellow	tawny	filemot	
Point	0	1	2	3	
IHC score	Score of positive cell proportion × Score of staining degree of positive cells

**Table 2:** TRMT6 and related clinical information were statistically analyzed by Chi-square test

	TRMT6 High	TRMT6 Low	*p*-value
Age (year)			1
> = 50	18	18	
< 50	20	20	
Gender			0.69161
Male	34	35	
Female	4	3	
BCLC stage			0.26674
A	13	20	
B	8	4	
C	16	14	
D	1	0	
Tumour-Node-Metastasis (TNM) stage			0.00006
I	5	17	
II	15	3	
III	7	12	
IV	11	6	
Primary tumor (T) classification			0.00191
T1	5	17	
T2	20	7	
T3	8	12	
T4	5	2	
Regional lymph node (N) classification			0.010701
N0	32	38	
N1	6	0	
Distant metastasis (M) classification			0.168716
M0	27	32	
M1	11	6	
Recurrence			0.22186
No	10	15	
Yes	28	23	
Portal vein tumor thrombus			0.236593
No	33	31	
Yes	5	7	
Microscopic tumor thrombus			0.118061
No	25	31	
Yes	13	7	

### TRMT6 promoted the proliferation of HCC cells and increased the proportion of S phase cells

**Fig. 2:**
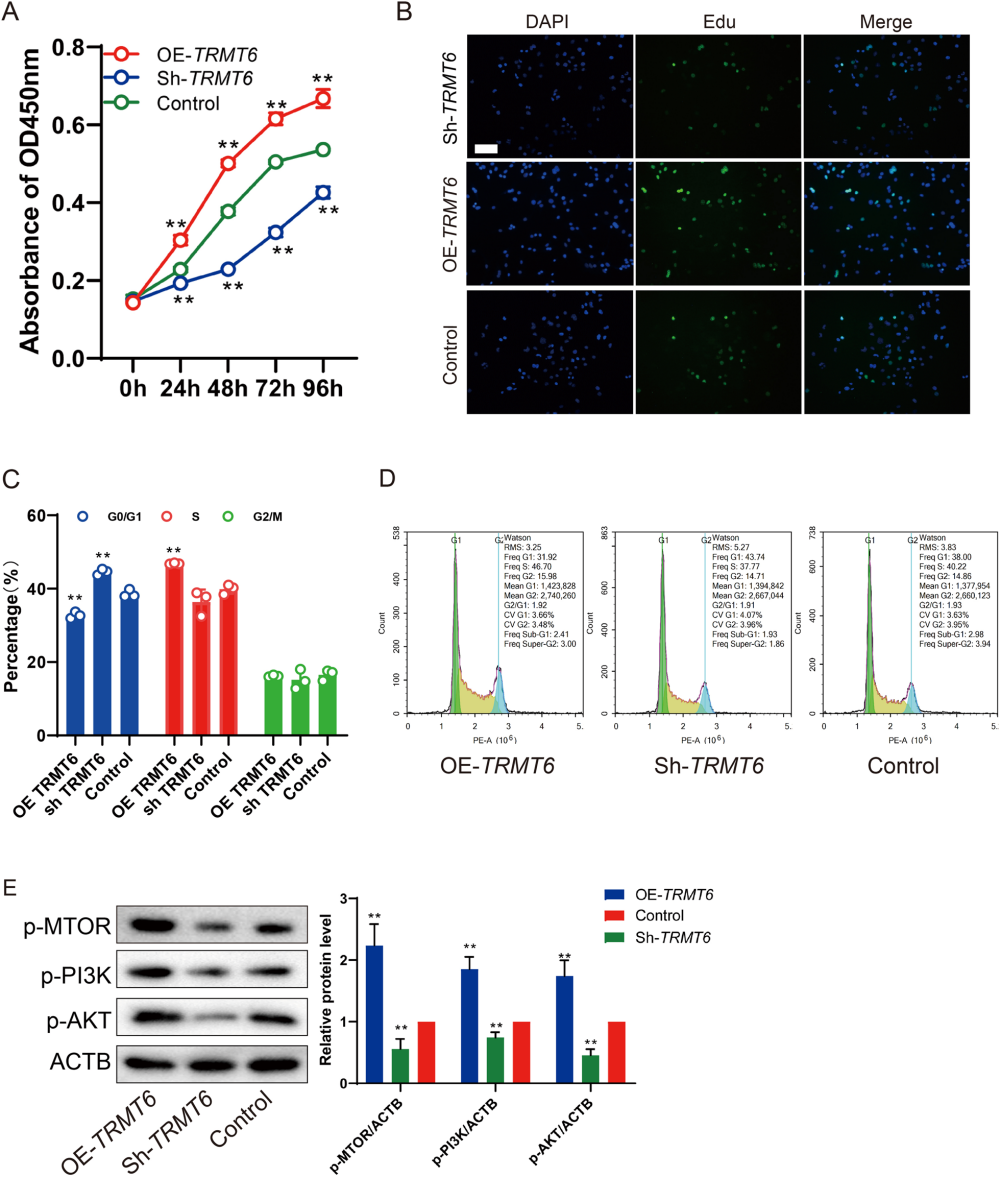
TRMT6 promoted the proliferation of HCC cells and increased the proportion of S phase cells. HepG2 cells were divided into control group, OE-*TRMT6* group, and sh*TRMT6* group. **A** Cell activity was used to detect by CCK-8 assay. **P < 0.01 vs control. **B** EDU cell proliferation detection was used to detect the number of EDU positive cells. **C**, **D** Effect of TRMT6 expression on HepG2 cell cycle distribution was detected by flow cytometry. **P < 0.01 vs control group. **E** Expression of p-PI3K, p-AKT and p-mTOR in HepG2 cells were detected by Western blot

Since TRMT6 is highly expressed in a variety of HCC cells and cancer tissues, and according to previous studies [21, 22], it is likely to be highly correlated with the cell proliferation of HCC cells and PI3K/AKT signal, so we will study whether TRMT6 regulates the proliferation of HCC cells in the following studies. HepG2 cells were divided into control group, OE-*TRMT6 *group, and sh*TRMT6* group after TRMT6 overexpression or interference. Transfection efficiency verification of OE-T*RMT6* or sh*TRMT6* is shown in Additional file 1: Figure S2. After inhibiting or overexpressing the expression of TRMT6 in HepG2 cells, CCK-8 assay was performed to detect the cell activity. The results showed that the cell activity in HepG2 cells decreased after interference with TRMT6, and the cell activity increased after TRMT6 overexpression, and showed time dependence (Fig. 2A). The staining results of EDU cell proliferation detection showed that the number of green stained cells (EDU positive cells) in HepG2 cells was increased after TRMT6 overexpression, and the number of EDU positive cells was decreased after interference with TRMT6, indicating that interference with TRMT6 could significantly inhibit the proliferation of HepG2 cells (Fig. 2B). Flow cytometry results showed that the proportion of G0/G1 phase cells in the sh*TRMT6* group was higher than that in control group, and the proportion of S phase cells was lower than that in control group (Fig. 2C, D). In addition, overexpression of TRMT6 showed the opposite result (Fig. 2C, D). The expression changes of phosphatidylinositol 3-kinase (PI3K), AKT and mammalian target of rapamycin (mTOR) in HepG2 cells were detected by Western blot, and the results showed that compared with the control group, overexpression of TRMT6 increased the expressions of p-PI3K, p-AKT and p-mTOR related to the PI3K/AKT/mTOR signaling pathway, and interference with TRMT6 decreased these proteins’ expressions (Fig. 2E). In conclusion, it can be found that TRMT6 overexpression can promote the proliferation of HCC cells, increase the number of S phase cells, and promote DNA synthesis. Moreover, changing the expression of TRMT6 will affect the related protein expressions of the PI3K/AKT signaling pathway.

### TRMT6 promotes cell proliferation through the PI3K/AKT signaling pathway

**Fig. 3:**
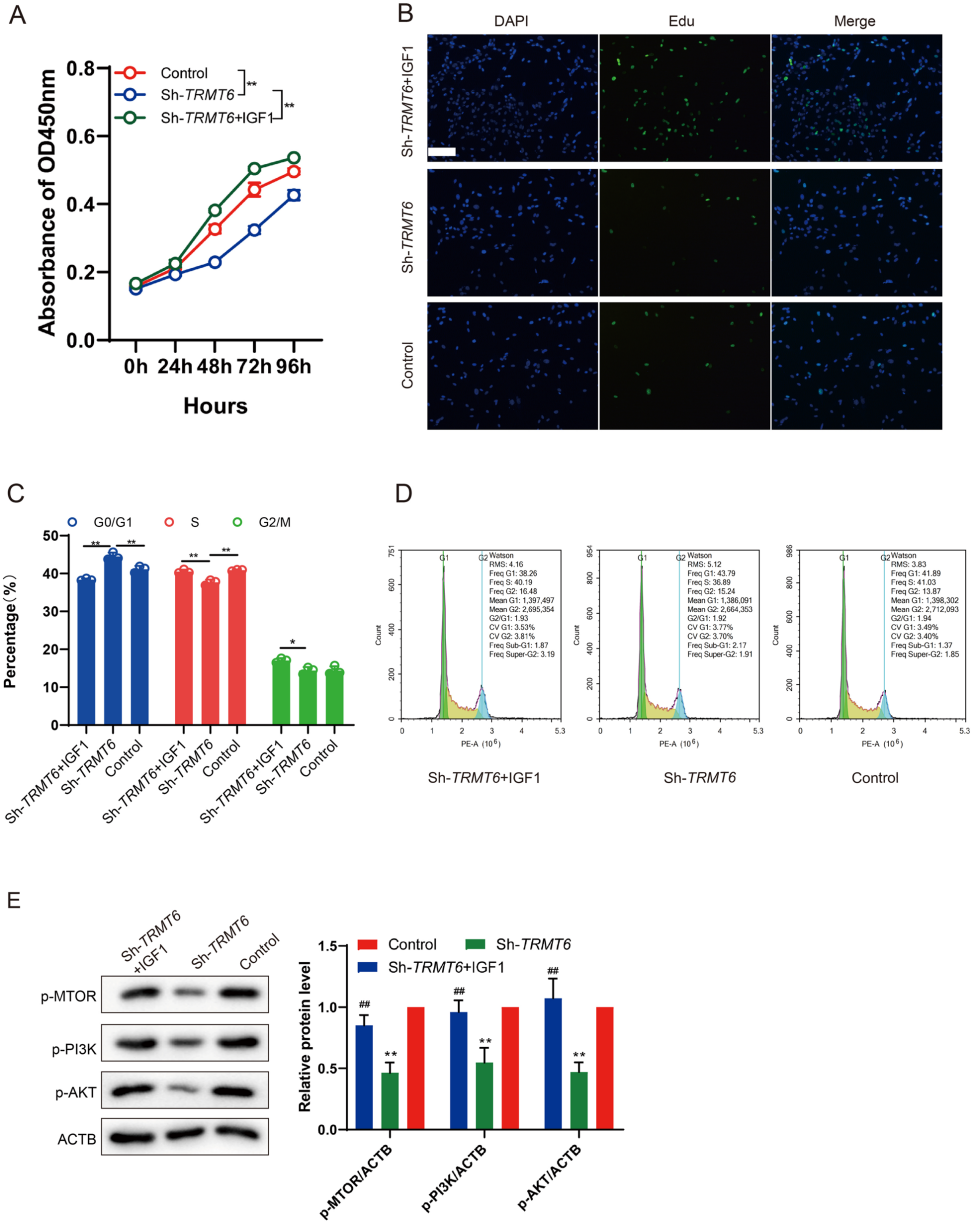
TRMT6 promotes cell proliferation by regulating the PI3K/AKT signaling pathway. HepG2 cells transfected with sh*TRMT6* were treated with PI3/AKT signaling pathway activator IGF1, and grouped into control group, sh*TRMT6* group and sh*TRMT6* + IGF1 group. **A** CCK-8 assay was used to detect the cell activity. **P < 0.01 vs control or sh*TRMT6* group. **B** EDU cell proliferation detection was used to detect the number of EDU positive cells. **C**, **D** Cell cycle distribution was detected by flow cytometry. *P < 0.05, **P < 0.01 vs control or sh*TRMT6* group. **E** Protein expressions of p-PI3K, p-AKT and p-mTOR in HepG2 cells were detected by Western blot

To further verify that TRMT6 regulates the proliferation of HepG2 cells by inhibiting the PI3K/AKT signaling pathway, HepG2 cells transfected with sh*TRMT6* were treated with PI3K/AKT signaling pathway activator IGF1, and were grouped into control group, sh*TRMT6* group and sh*TRMT6* + IGF1 group. The results of CCK-8 detection showed that the cell activity in HepG2 cells was decreased after the inhibition of TRMT6 level in the sh*TRMT6* group, and the addition of IGF1 restored HepG2 cell proliferation in the sh*TRMT6* + IGF1 group (Fig. 3A).

The number of EDU positive cells in the sh*TRMT6* group was decreased when compared to control group, and the number of EDU positive cells was increased after the addition of IGF1, indicating that TRMT6 could inhibit the proliferation of HepG2 cells by regulating the PI3K/AKT signaling pathway (Fig. 3B). Flow cytometry results showed that the proportion of G0/G1 phase cells in the sh*TRMT6* group was higher than that in control group, and the proportion of S phase cells was lower than that in control group (Fig. 3C, D). Furthermore, the addition of IGF1 reversed the effect of TRMT6 interference (Fig. 3C, D). We also found that when compared to control group, p-PI3K, p-AKT and p-mTOR protein expressions in the sh*TRMT6* group were decreased, and the treatment of IGF1 increased the protein expressions (Fig. 3E). According to the above results, it was found that interference with TRMT6 reduced the expression of proteins related to the PI3K/AKT signaling pathway, and significantly increased cell proliferation by activating the signaling pathway. Moreover, activator treatment proved that TRMT6 indeed regulated cell proliferation through the PI3K/AKT signaling pathway.

### The effect of TRMT6 on HCC tumor growth

**Fig. 4:**
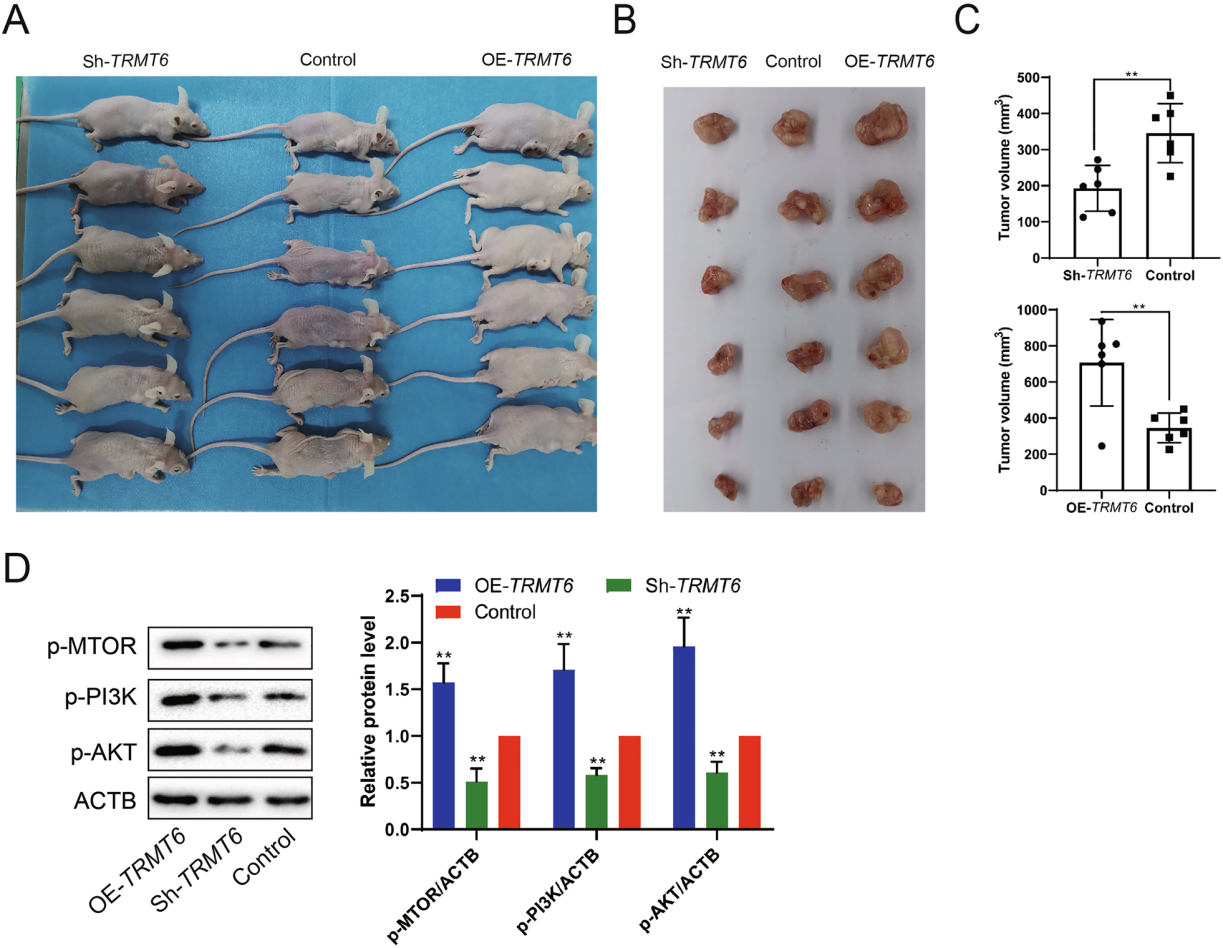
Effect of TRMT6 on HCC tumor growth. The mice were divided into the following groups: control group, OE-*TRMT6* group and sh*TRMT6* group. **A**, **B** After 4 weeks, the tumor growth of mice in each group was photographed. **C** Tumor volume of each group was statistically analyzed. *P < 0.05, **P < 0.01 vs control group. **D** Protein expressions of p-PI3K, p-AKT and p-mTOR in tumor tissues were detected by Western blot

Next, different groups of cells were injected subcutaneously in nude mice to construct the transplanted tumor model. The mice were divided into the following groups: control group, OE-*TRMT6* group and sh*TRMT6* group. After 4 weeks, the tumor growth of mice in each group was detected (Fig. 4A, B). The results showed that the tumor size was the largest in the TRMT6 overexpression group and the smallest in the interference group (Fig. 4C). In addition, we also detected the protein expression of TRMT6 downstream pathway-related proteins in tumor tissues, and the results showed that overexpression of TRMT6 could increase the expressions of p-PI3K, p-AKT and p-mTOR, while interference with TRMT6 had the opposite effect (Fig. 4D). The above in vivo experiments indicated that overexpression of TRMT6 promoted tumor growth and was related to PI3K/AKT signaling pathway.

## Discussion

Several studies have reported the impact of RNA methylation on the occurrence and prognosis of liver cancer, which has attracted extensive attention [23, 24]. m^1^A modification can regulate mRNA translation and tRNA stability, but its specific functions and mechanisms in HCC and other tumor progression remain to be explored [16, 25–27]. It has been shown that highly active tRNA-modifying enzymes in malignant tumor cells can highly modify tRNA with normal structure to produce abnormal tRNA, and a large number of modified nucleosides can be produced during the metabolism of the latter [28]. In addition, there is an increasing interest in the study of RNA methylation modification N1 methyladenosine (m^1^A). Currently, urine levels of modified nucleosides have been used for diagnosis, efficacy evaluation, and prognosis monitoring of a variety of tumors, including m^1^A [29]. Recent studies have found that the expressions of several known m^1^A regulatory enzymes are abnormally increased in HCC and also associated with poor prognosis in HCC patients [30]. Silencing of the m^1^A demyltransferase ALKBH3 inhibited the AKT1S1 and ERBB2 expressions, which are the key molecule of PI3K/AKT/mTOR and ErbB signaling pathway, suggesting that m^1^A modification plays an important role in the development of HCC [30]. However, its specific functions and mechanisms in tumor initiation and progression such as HCC need to be further explored.

According to the analysis of TCGA database, Zhao et al*.* found that TRMT6 was highly expressed in a variety of cancers, including liver cancer [30]. Wang et al*.* found that *TRMT6* mRNA level was significantly correlated with the pathological characteristics and prognosis of HCC patients [31]. Yeon et al*.* reported that *TRMT6* gene has been shown to be an oncogene associated with colorectal adenocarcinoma, and its frameshift mutation may lead to gene inactivation and inhibit the occurrence and development of tumors [32]. In this study, the expression of TRMT6 in HCC tissues was statistically analyzed using the LIHC data set in the TCGA database, and the adjacent tissues were used as controls. At the same time, survival analysis was performed to observe whether TRMT6 correlated with the survival rate of patients, and the results showed that TRMT6 was closely associated with the progression and prognosis of HCC. We also verified the analysis and clinical results of previous studies by tissue microarray assay, and found that TRMT6 overexpression may be a significant risk factor affecting the prognosis of HCC. In addition, the results of qRT-PCR and Western blot showed that the expression level of TRMT6 was relatively high in HCC cell lines compared with the normal hepatocytes LX-2.

In the current research progress, there are still few studies on the mechanism of m^1^A-related regulatory enzymes and carcinogenesis. According to the study of Zhao et al*.* the GO analysis found that m^1^A regulatory enzymes were associated with proliferation-related ErbB and mTOR signaling pathways [30]. The PI3K/AKT signaling pathway is involved in the regulation of cell growth and proliferation and shows structural activation in different cancer types [33, 34]. PI3K activity is stimulated by a variety of oncogenes and growth factor receptors, so activation of PI3K signaling pathway is a key factor in cancer. Therefore, this study took this as an entry point to speculate that TRMT6 may regulate the PI3K/AKT signaling pathway in which mTOR is located, thereby regulating the proliferation of liver cancer cells. In this study, the role of TRMT6 on the growth of HCC cells was first studied in vitro. The results showed that interfering with TRMT6 could inhibit the proliferation of cancer cells, the number of S phase cells and the protein expressions of p-PI3K, p-AKT and p-mTOR. In addition, the addition of PI3K/AKT signaling activator could reverse this phenomenon, suggesting that TRMT6 indeed regulates cell proliferation through the PI3K/AKT signaling pathway. Then, we conducted in vivo experiments to verify the effects of overexpression and interference of TRMT6 on tumor growth.

At present, the effect of m^1^A modification on tumor is still in the stage of investigation, and it is not clear whether m^1^A methyltransferases such as TRMT6/61A can play a role by directly regulating the translation of target mRNA. Therefore, this paper will mainly conduct a preliminary study on the effect of m^1^A modification on the development of HCC, and subsequent studies need to be adjusted according to the specific progress.

## Supplementary Information

**Additional file 1: Figure S1.** Spearman’s rank correlation coefficient analysis was performed between the scores of Ki67 and TRMT6.

**Additional file 2: Figure S2.** HepG2 cells were divided into control, OE-*TRMT6*, and sh*TRMT6* after TRMT6 overexpression or interference. Transfection efficiency verification of OE-T*RMT6* or sh*TRMT6* was detected by qRT-PCR and western blot assays.

## Data Availability

The data sets used and/or analyzed during the current study are available from the corresponding author on reasonable request.
