# Supplementary figures and images for: TRMT6 promotes hepatocellular carcinoma progression through the PI3K/AKT signaling pathway
Source: Eur J Med Res. 2023 Jan 27;28:48. doi: 10.1186/s40001-022-00951-1 (PMC9881333; doi:10.1186/s40001-022-00951-1)

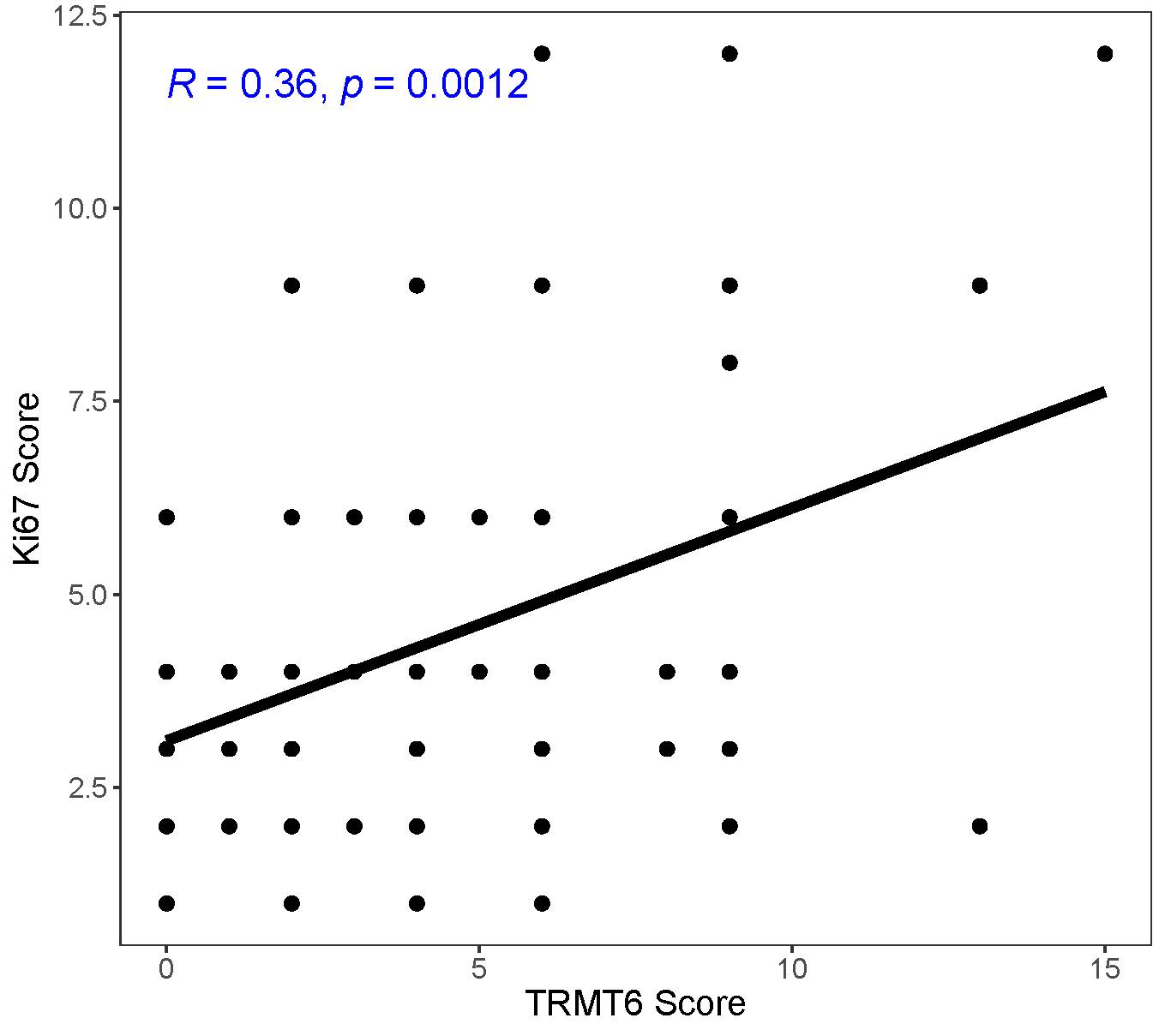

Supplement: Supplementary file 1 — Additional file 1: Figure S1. Spearman’s rank correlation coefficient analysis was performed between the scores of Ki67 and TRMT6. [file 40001_2022_951_MOESM1_ESM.jpg]

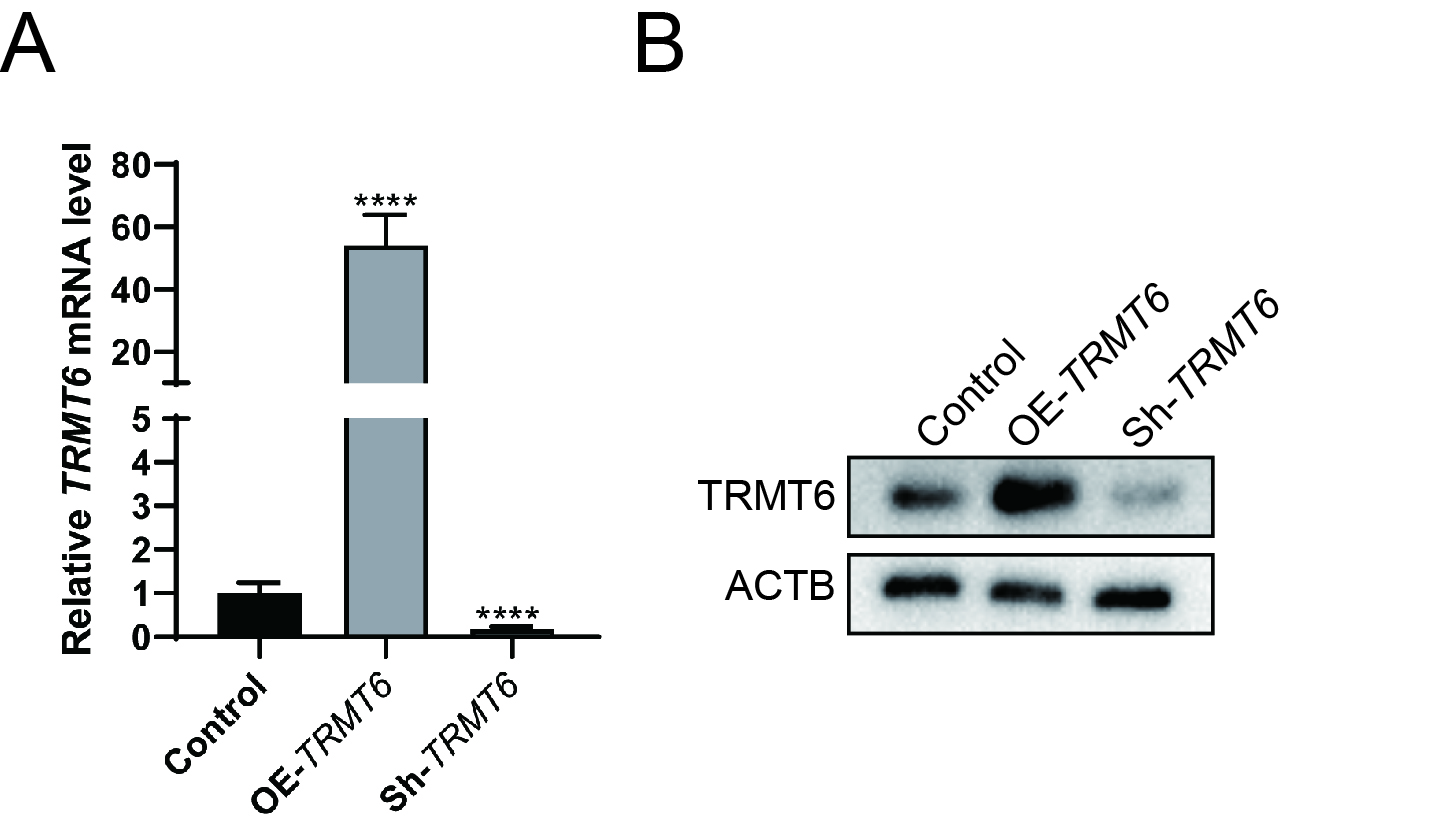

Supplement: Supplementary file 2 — Additional file 2: Figure S2. HepG2 cells were divided into control, OE-TRMT6, and shTRMT6 after TRMT6 overexpression or interference. Transfection efficiency verification of OE-TRMT6 or shTRMT6 was detected by qRT-PCR and western blot assays. [file 40001_2022_951_MOESM2_ESM.jpg]
